# Circulating microRNAs as biomarkers for type 1 diabetes mellitus

**DOI:** 10.1186/1758-5996-7-S1-A206

**Published:** 2015-11-11

**Authors:** Tais Silveira Assmann, Marcia Khaled Puñales Coutinho, Balduíno Tschiedel, Luis Henrique Canani, Daisy Crispim

**Affiliations:** 1 Hospital de Clínicas de Porto Alegre, Porto Alegre, Brazil

## Background

Type 1 diabetes mellitus (T1D) is usually diagnosed when >80% of the pancreatic beta-cells are destroyed by the immune system. The autoimmune destruction of these cells is slow, providing a potentially long lag of time to identify individuals at risk, and maybe prevent T1D development. We can predict T1D development through the determination of some islet autoantibodies. However, these antibodies appear fairly late in the course of T1D, not being ideal biomarkers of the initial destruction of beta-cells. Therefore, new biomarkers are needed to improve the identification of at risk-individuals. MicroRNAs (miRNAs) are a class of small noncoding RNA molecules that negatively regulate gene expression by inducing target mRNA cleavage or by inhibiting protein translation. Changes in their expression were described in several pathological conditions, including autoimmune diseases. Circulating miRNAs are attractive biomarker candidates as they can be easily collected, are stable under different storage conditions and can be measured using specific assays.

## Objective

To investigate circulating candidate miRNAs as potential biomarkers for T1D diagnosis.

## Materials and methods

We analyzed 25 T1D patients (13: <5 yrs. of diagnosis, and 12: >5 yrs. of diagnosis) and 20 age- and gender-matched nondiabetic controls. Expressions of 48 miRNAs were investigated in the plasma using Stem-loop RT-PreAmp Real-time PCR and TaqMan Low Density Array cards (Life Technologies). Array data was analyzed using Symphony (Life Technologies) and SPSS 18.0 programs.

## Results

Seventy-seven percent (37/48) of the miRNAs analyzed were detected in plasma of the sample, some described as being involved in immune regulation or beta-cell function. Among these 37 miRNAs, 35.1% (13/37) were differentially expressed between controls and T1D patients with <5 yrs. of diagnosis: 15.4% (2/13) miRNAs were 2-5 n-fold downregulated (miR-93* and miR-146a) while 84.6% (11/13) miRNAs were 2-40 n-fold upregulated (miR-101, miR-200a, miR-148b, miR-210, miR-155, miR-320, miR-103, miR-145, miR-21*, miR-126, miR-148a) in T1D patients. On the other hand, no differences were detected between controls and T1D patients with >5 yrs. of diagnosis.

## Conclusion

Our data demonstrate that some circulating miRNAs are differentially expressed in T1D patients in the first yrs. of the diagnosis. Ongoing studies will further explore the role of these miRNAs as novel biomarkers for T1D prediction.

